# *Chlamydia trachomatis* Test-of-Cure Cannot Be Based on a Single Highly Sensitive Laboratory Test Taken at Least 3 Weeks after Treatment

**DOI:** 10.1371/journal.pone.0034108

**Published:** 2012-03-28

**Authors:** Nicole H. T. M. Dukers-Muijrers, Servaas A. Morré, Arjen Speksnijder, Marianne A. B. van der Sande, Christian J. P. A. Hoebe

**Affiliations:** 1 Department of Sexual Health, Infectious Diseases and Environmental Health, Geleen, South Limburg Public Health Service, South Limburg, The Netherlands; 2 Department of Medical Microbiology, School of Public Health and Primary Care (CAPHRI), Maastricht University Medical Center (MUMC+), Maastricht, The Netherlands; 3 VU University Medical Center, Medical Microbiology and Infection Prevention, Amsterdam, The Netherlands; 4 Institute of Public Health Genomics, Department of Genetics and Cell Biology, Research Institutes CAPHRI and GROW, Faculty of Health, Medicine and Life Sciences, University of Maastricht, Maastricht, The Netherlands; 5 Public Health Laboratory, Health Service Amsterdam, The Netherlands; 6 Epidemiology and Surveillance, Centre for Infectious Diseases Control, National Institute for Public Health and the Environment, Bilthoven, The Netherlands; 7 Julius Centre, University Medical Center Utrecht, Utrecht, The Netherlands; Auburn University, United States of America

## Abstract

Current test-of-cure practice in patients with *Chlamydia trachomatis* (Ct) infection is to confirm cure with a single test taken at least 3 weeks after treatment. Effectiveness of single-time-point testing however lacks a scientific evidence basis and the high sensitivity of laboratory assays nowadays in use for this purpose may compromise the clinical significance of their results. Prospectively following 59 treated Ct infections, administering care as usual, the presence of Ct plasmid DNA and rRNA was systematically assessed by multiple time-sequential measurements, i.e. on 18 samples taken per patient during 8 weeks following treatment with a single dose of 1000 mg Azythromycin. A high proportion (42%) of Ct infections tested positive on at least one of the samples taken after 3 weeks. Patients' test results showed substantial inter-individual and intra-individual variation over time and by type of NAAT used. We demonstrated frequent intermittent positive patterns in Ct test results over time, and strongly argue against current test-of-cure practice.

## Introduction

Current treatment practice in *Chlamydia trachomatis* (Ct) infections is challenged by a growing concern over the efficacy of Azythromycin, currently the recommended antibiotic treatment [1], [2], [3]. Data indicating sub-optimal effectiveness were presented at the recent meeting of the International Society for Sexually Transmitted Diseases Research (Québec, Canada July 2011) [4]. It was also noted that assessment of actual treatment failure is hampered by the difficulty to differentiate between re-infection and antibiotic resistance in vivo. To confirm clearance of Ct infection, and thus deliver a proof of cure, clinicians can apply a single time-point test-of-cure, using nucleic acid amplification assays (NAAT). There are currently no data available on the optimal timing of testing for cure; generally, testing no sooner than 3 weeks and no later than 3 months after treatment is recommended [1], [2]. Current guidelines by the Centers for Disease Control and Prevention, advocate restricted use of a test-of-cure, i.e. only when a patient is pregnant, therapeutic compliance is questioned, symptoms persist, or re-infection is suspected [1]. Data on the actual use of a test-of-cure are scarce, although results from a recent large-scale US study among women suggested inadequate adherence to current testing guidelines [5]. Nevertheless, there are data suggesting that test-of-cure practices are by no means uncommon. In The Netherlands for example, 11% of men and 27% of women with an initial Ct-positive test were retested within the first 3 months (unpublished South Limburg laboratory registry data). In the US, 21% of all repeat Ct tests in women between 15 and 25 years of age who were enrolled in commercial health plans and had two or more Ct tests, were performed within the second and third month (personal communication J. Heijne, MSc. 2012 University of Bern, Bern, Switzerland). It should be noted though, that in this latter dataset the result of the initial Ct test and reason for testing were unknown [6].

Effectiveness of the current test-of-cure practices using single-time-point testing however lacks a scientific evidence basis. The current practice of using highly sensitive NAAT for test-of-cure practice has several well-known shortcomings [7]. A positive result may reflect treatment failure with persistent infection, but may also reflect resolved infection by detecting the mere presence of ribosomal RNA debris and non-viable Ct DNA [8]. Other possible explanations include detection of re-infection or transient Ct DNA after sex with an infected partner. Even in persistent infection, a positive test may be preceded by a negative post-treatment NAAT [9], [10]. The clinical conundrum of delivering a proof of cure is further addressed here. We explored consistency in individual test-of-cure results by NAAT in hypothetical clinical situations, taken to reflect actual test-of-cure practices. In a cohort of Ct treated patients, we systematically assessed the presence of Ct plasmid DNA and rRNA by multiple time-sequential measurements on 18 samples per Ct infection taken during 8 weeks following treatment with a single dose of 1000 mg Azythromycin.

## Results

**Figure 1:**
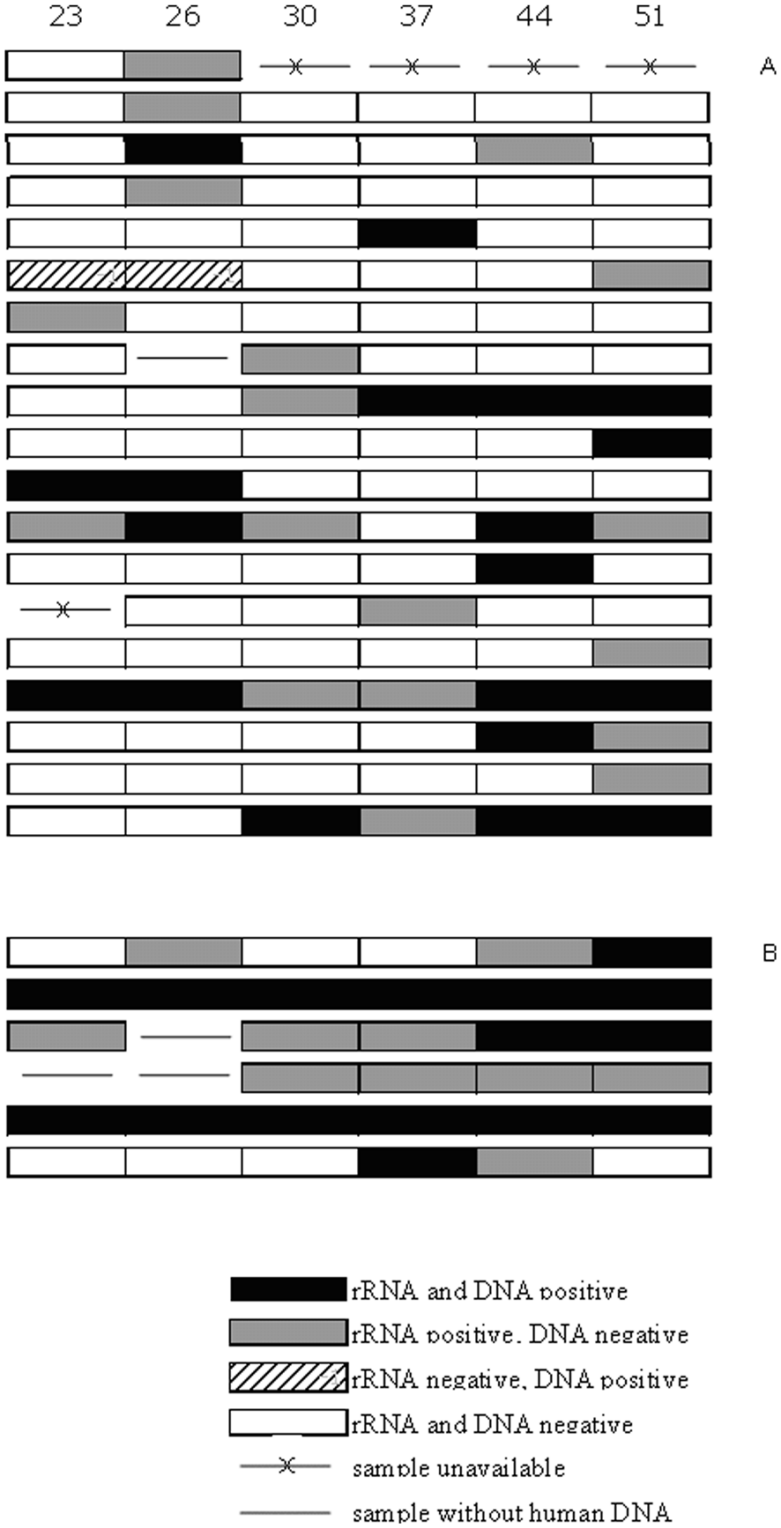
Cervicovaginal (a) and anorectal (b) *Chlamydia trachomatis* rRNA and DNA detection between 23 and 51 days post directly observed Azythromycin treatment. Each row represents a cervicovaginal or anorectal Ct infection. Twenty five infections (out of 59) had at least one positive sample between 23 and 51 days post-treatment and these 25 infections are displayed here. Self taken swabs were tested for Ct rRNA (TMA, Tigris; GenProbe, San Diego, US) and plasmid DNA (real-time in house PCR; Amsterdam, the Netherlands) [12].

Taking a test-of-cure at 23, 26, 30, 37, 44 and 51 days post-treatment, 14%, 20%, 16%, 17%, 22% and 24%, respectively, of the 59 Ct infections tested positive for rRNA and/or DNA. Overall, 42% (n = 25) of the Ct infections tested positive on at least one of the samples taken between 23 and 51 days; 42% (n = 25) tested positive for rRNA and 27% (n = 16) for DNA. The test results of these 25 infections showed substantial inter-individual and intra-individual variation over time and by type of NAAT used, as is shown in Fig. 1. Most infections tested positive intermittently. Inadequate self-sampling seemed unlikely as a possible explanation for intermediary Ct-negative tests as human DNA was detected in the majority of Ct-negative samples; samples from 3 Ct infections did not contain human DNA. One cervicovaginal and 4 anorectal Ct infections consistently tested rRNA-positive, of which 2 anorectal infections also tested Ct DNA-positive in all samples taken between 23 and 51 days post treatment. In total, 66% of the 59 Ct infections demonstrated test results that were consistently negative (n = 34) or positive (n = 5) for Ct rRNA and/or DNA between 23 and 51 days post treatment.

## Discussion

Given the pattern of intermittent positive results and substantial variability between different types of NAAT used, it seems unjustified to interpret a single positive NAAT taken between 3 to 8 weeks post-treatment as treatment failure/persistence or (re-)infection. Likewise, the clinical significance of a single negative NAAT may be debatable. Our results reinforce the known difficulties in establishing a proof of cure. A positive NAAT may occur due to treatment failure, but also due to other causes. A negative NAAT may indicate cleared infection, but may also reflect persistent infection. Sexual history is not a reliable tool to confirm or rule out new Ct infection, and at present there are no specific laboratory tests that allow clinicians to reliably distinguish between treatment failure/persistence or resistance. It is a real, perhaps even insurmountable, challenge to overcome this problem.

Yet, in current clinical practice a single time-point test-of-cure by NAAT is taken to reflect a patient's Ct clearance status. Alongside the positive aspects, i.e. reassurance of worried patients and, sometimes, more adequate treatment, the negative consequences of current test-of-cure practices - including unnecessary antibiotic treatment and laboratory testing - should not be overlooked. Considering that clinical significance of a positive test-of-cure by NAAT is unknown, sequential rather than single-time point testing may not solve the problem either. The individual and public health gains and cost-effectiveness of test-of-cure practices by NAAT remain to be established. Treatment practice may need to focus more on improving compliance of the patient and their partners in treatment with state-of-the-art regimens [11] and increasing the proportion of patients re-tested after 3 to 12 months, in accordance with current recommendations [1], [2].

## Methods

### Ethics statement

The study was ethically approved by the Medical Ethics Committee at the Free University of Amsterdam (UvA:2009/154,CCMO The Hague:NL28609.029.09). Clinicaltrials.gov Identifier: NCT01448876.

### Study population and setting

By convenience sampling at our outpatient STD clinic (South Limburg, the Netherlands), 46 non-pregnant women and 6 men were included, contributing 44 cervicovaginal and 15 anorectal infections; 7 women contributed infections from both anatomic sites. Participants provided a total of 1016 self-taken cervicovaginal and/or anorectal swabs taken over a period from 0 until 51 days post-treatment, at pre-defined points of time. Overall, 94% of samples were delivered according to schedule, and were laboratory tested. All patients were negative for HIV, gonorrhea and *Lymphogranuloma venerum* (the latter was tested in anorectal Ct-positive samples only). Usual care recommending abstinence or safe sex for one week and providing treatment for steady partners was administered [1], [2]. Participants provided written informed consent.

### Analyses

Swabs were tested for Ct rRNA (TMA, Tigris; GenProbe, San Diego, US) and plasmid DNA (real-time in house PCR; Amsterdam, the Netherlands) [12]. All Ct-negative samples taken at the 2 time points preceding and following a Ct-positive sample between 16 and 51 days post-treatment were re-tested for human DNA to rule out that a negative result was due to inadequate sampling. Test results of samples not containing human DNA were considered missing in analyses. The proportion of rRNA and/or DNA-positive samples was assessed during 23 and 51 days post-treatment

## References

[1] Centers for Disease Control and Prevention (CDC) (2010). STD Treatment Guidelines.. MMWR Recomm Rep.

[2] British Association for Sexual Health and HIV (BASHH) (2010). *Chlamydia trachomatis* UK Testing Guidelines.

[3] Handsfield H (2011). Questioning Azithromycin for Chlamydial Infection.. Sex Trans Dis.

[4] Drummond F, Ryder N, Wand H, Guy R, Read P (2011). Is azithromycin adequate treatment for asymptomatic rectal chlamydia?. Int J STD AIDS.

[5] Hoover K, Tao G, Body B, Nye M, Kent C (2011). Suboptimal repeat testing of women with positive chlamydia tests in the usa, 2008–2010. 20th ISSTDR in conjunction with BASHH Congress in Québec, Canada, 10–13 July 2011(O1-S01.04).. Sex Transm Infect.

[6] Heijne J, Althaus C, Herzog S, Tao G, Kent C (2011). Estimating the rate of annual chlamydia screening uptake in us women. 20th ISSTDR in conjunction with BASHH Congress in Québec, Canada, 10–13 July 2011 (O1-S01.05).. Sex Transm Infect.

[7] Hadgu A, Sternberg M (2009). Reproducibility and specificity concerns associated with nucleic acid amplification tests for detecting Chlamydia trachomatis.. Eur J Clin Microbiol Infect Dis.

[8] Morré SA, Sillekens PT, Jacobs MV, de Blok S, Ossewaarde JM (1998). Monitoring of Chlamydia trachomatis infections after antibiotic treatment using RNA detection by nucleic acid sequence based amplification.. Mol Pathol.

[9] Horner P (2006). The case for further treatment studies of uncomplicated genital Chlamydia trachomatis infection.. Sex Transm Infect.

[10] Suchland RJ, Sandoz KM, Jeffrey BM, Stamm WE, Rockey DD (2009). Horizontal Transfer of Tetracycline Resistance among *Chlamydia* spp. In Vitro. Antimicrob agents and chemother..

[11] Geisler WM, Mena L, Taylor SN, Batteiger BE, Thurman A (2011). Safety and efficacy of WC2031 vs vibramycin for the treatment of uncomplicated urogenital *Chlamydia trachomatis* infection 20th ISSTDR in conjunction with BASHH Congress in Québec, Canada, 10–13 July 2011 (O3-S4.03). Sex Transm Infect.

[12] Catsburg A, Savelkoul PMH, Vliet A, Algra J, Vandenbroucke-Grauls CMJE, Chernesky M, Caldwell H, Christiansen G (2006).

